# N-Doped Carbon Nanowire-Modified Macroporous Carbon Foam Microbial Fuel Cell Anode: Enrichment of Exoelectrogens and Enhancement of Extracellular Electron Transfer

**DOI:** 10.3390/ma17010069

**Published:** 2023-12-22

**Authors:** Ke Liu, Zhuo Ma, Xinyi Li, Yunfeng Qiu, Danqing Liu, Shaoqin Liu

**Affiliations:** 1 School of Material Science and Chemical Engineering, Harbin University of Science and Technology, Harbin 150040, China; 2 Harbin Institute of Technology, School of Life Science and Technology, Harbin 150001, China; 3 Key Laboratory of Microsystems and Microstructures Manufacturing, Harbin Institute of Technology, School of Medicine and Health, Harbin 150080, China

**Keywords:** microbial fuel cells, anode, extracellular electron transfer, hierarchical porous structure, exoelectrogen enrichment

## Abstract

Microbial fuel cell (MFC) performance is affected by the metabolic activity of bacteria and the extracellular electron transfer (EET) process. The deficiency of nanostructures on macroporous anode obstructs the enrichment of exoelectrogens and the EET. Herein, a N-doped carbon nanowire-modified macroporous carbon foam was prepared and served as an anode in MFCs. The anode has a hierarchical porous structure, which can solve the problem of biofilm blockage, ensure mass transport, favor exoelectrogen enrichment, and enhance the metabolic activity of bacteria. The microscopic morphology, spectroscopy, and electrochemical characterization of the anode confirm that carbon nanowires can penetrate biofilm, decrease charge resistance, and enhance long-distance electron transfer efficiency. In addition, pyrrolic N can effectively reduce the binding energy and electron transfer distance of bacterial outer membrane hemin. With this hierarchical anode, a maximum power density of 5.32 W/m^3^ was obtained, about 2.5-fold that of bare carbon cloth. The one-dimensional nanomaterial-modified macroporous anodes in this study are a promising strategy to improve the exoelectrogen enrichment and EET for MFCs.

## 1. Introduction

MFCs are an emerging bioelectrochemical technology that utilize microorganisms attached to the surface of an anode to convert chemical energy stored in organic waste into electrical energy [1,2,3]. At the same time, they can treat organic pollutants in wastewater using aeration, without consuming additional energy [4,5,6]. The anode of MFCs is an important site for microbial adhesion and EET. Traditional current collectors such as carbon cloth (CC), carbon felt, and stainless steel mesh can all be used as anodes for MFCs [7,8,9,10]. However, due to their poor biocompatibility, they are unable to enrich exoelectrogens, resulting in unsatisfactory battery performance [11,12]. Researchers have found that modifying these collectors with nanocarbon materials, nanometals or metal oxides, and polymers can effectively promote the enrichment of exoelectrogens, as well as the uptake and transfer of electrons [13,14,15,16]. However, many studies have confirmed that after long-term operation, the nanomaterial-modified current collector still faces pore structure blockage [17,18]. As a result, the internal bacterial metabolism is hindered, causing a large number of bacterial deaths, which in turn affects the long-term power generation and sewage treatment performance of MFCs.

The porous anode surface can provide more attachment surfaces for bacteria, induce them to produce conductive conduits, enrich direct electron transfer pathways, reduce the internal resistance of MFCs, and eventually improve output power [19,20]. Recently, researchers have designed microporous, mesoporous, and macroporous hierarchical composite N-doped carbon-based MFC anode materials using reverse opal structures [21]. The introduction of a 3D pore structure and N species can improve the hydrophilicity of the anode; promote the adhesion, mass transfer, and EET of the biofilm; and achieve higher output power. The start-up time was only 2.9 days, and the volume power density reached 6.38 W/m^3^ when using actual beer wastewater as the anode solution, with a COD removal rate of 84.33%. Carbonized biomass materials can easily obtain macroporous MFC anodes. For example, Karthikeyan et al. obtained macroporous anodes from high-temperature carbonized corn straw as raw materials and found that in the early stages, the internal and external pores of the anode could promote the attachment of exoelectrogens [22]. However, during long-term operation, it was found that internal pores with size of 2–7 μm may be blocked by biofilms, resulting in the death of a large number of exoelectrogens inside. Usually, common exoelectrogens such as *Geobacter* and *Shewanella* have a diameter of 1–2 μm. They are not suitable for colonization on the surface of nanostructures with small pore sizes, and the thickness of mature biofilms on the anode surface can reach tens of micrometers [23]. In theory, it is possible to inhibit biofilm blockage at least at the scale of tens or hundreds of micrometers, ensuring the mass transfer and removal of metabolized waste during long-term test. Therefore, it is necessary to develop high-performance macroporous-based MFC anode materials.

It is worth noting that a single increase in macroporous structure can promote the adhesion of biofilms and reduce blockage, but a lack of surface nanostructures can have a negative impact on the interface interaction between microorganisms and anodes [24,25]. To address this issue, researchers have used nanomaterials to modify macroporous anodes. For example, Huidong Li et al. obtained 3D macroporous MFC anodes modified with carbonized metal–organic framework (MOF) nanocrystals through high-temperature carbonization of gluten-loaded MOF materials [26]. Carbonized MOF nanocrystals have abundant mesoporous channels, which are conducive to promoting the excretion of flavin and synergistically promote EET by combining with the cytochrome C of the bacterial outer membrane. After long-term operation, the microorganisms inside the anode still maintain high metabolic activity, with a maximum power density of 11.21 W m^−3^, which is higher than that of the reported single macroporous anode material. Biomass can also be coated with precursors such as polypyrrole (PPy) and polydopamine (PDA), and N-doped carbon species can be introduced on the surface of macropores through high-temperature carbonization. It was found that pyrrolic N species can promote the adsorption of hemin on their surface, shorten the spatial distance, and promote EET. Through in-depth analysis of the direct electron transfer (DET) and mediated electron transfer (MET) mechanisms of EET, it was found that most of the reported nanoparticle-modified macroporous anodes lack long-range electron transfer pathways, which is not conducive to receiving electrons from remote bacteria [27]. Therefore, it is necessary to introduce one-dimensional (1D) nanowires on the surface of the macroporous anode to endow it with rich electron transfer pathways, increase the physical contact sites between the biofilm and the anode, and synergistically promote bacterial adhesion and EET [28].

In this study, melamine foam was carbonized to prepare a N-doped carbon macroporous carrier, and PPy nanowires were electrodeposited on its surface, which were further carbonized into nanowires rich in pyrrolic N (denoted as NC@CMF). This hierarchical porous structure can provide a large number of sites for bacterial reproduction. Meanwhile, N atoms on the carbon skeleton can shorten the electron transfer distance, generate local positively charged domains, and promote bacterial attachment and charge transfer. Therefore, the NC@CMF anode shows a higher output voltage (623 mV) and power density (5.32 W/m^3^) than bare CC.

## 2. Materials and Methods

### 2.1. Materials

The pyrrole was bought from Macklin Biochemical Co., Ltd. (Shanghai, China). Na_2_CO_3_, NaClO_4_, Na_2_HPO_4_·12H_2_O, NaH_2_PO_4_·2H_2_O, NH_4_Cl, KCl, CH_3_COONa, and K_3_[Fe(CN)_6_] were acquired from Aladdin Co., Ltd. (Shanghai, China). CC was purchased from Toray Industries, Inc. (Shanghai, China). Cation exchange membranes (1201) were bought from Grion Environmental Technology (Hangzhou, China). Power Soil DNA Isolation Kit was bought from Qiagen (Hilden, Germany).

### 2.2. Characterization

The surface structure of the materials was observed using scanning electron microscopy (SEM) (Quanta FEG; Thermo Fisher Scientific Inc., Waltham, MA, USA). The phase analysis and molecular structure of anodes were characterized using X-ray diffraction (XRD) (Rigaku D/Max 2500 PC; Rigaku Corporation, Tokyo, Japan), Raman spectroscopy (Raman) (inVia-Reflex03040405; Renishaw, London, UK), and energy-dispersive spectroscopy (EDS) (Quanta FEG; Thermo Fisher Scientific Inc., Waltham, MA, USA). The element composition and content of N species of materials were obtained with X-ray photoelectron spectroscopy (XPS) using a K−Alpha anode (Thermo Scientific, Waltham, MA, USA). The morphology of biofilms was characterized via SEM. Before SEM observation, the bacteria were fixed by paraformaldehyde and dehydrated using various ethanol solutions. The survival status of the biofilms on anode were researched using a confocal laser scanning microscope (CLSM) (LSM 880 NLO with Fast Airyscan, Carl Zeiss AG, Oberkochen, Germany).

### 2.3. Fabrication of CMF

The commercially available melamine foam (2 cm × 2 cm × 2 cm) was cleaned with ethanol and deionized water with ultrasonication, and carbonized at 900 °C for 1 h in N_2_ atmosphere to obtain CMF.

### 2.4. Fabrication of NC@CMF

The amperometric method was used to deposit PPy on CMF in a three-electrode system, which contained a working electrode of CMF (1 cm × 1 cm × 1 cm), a reference electrode of Ag/AgCl, and a counter electrode of platinum foil. In a typical electrochemical oxidation polymerization process, CMF was placed into 50 mL of electrolyte solution, which consisted of 1 g Na_2_CO_3_, 0.5 g NaClO_4_, and 500 μL of pyrrole. A constant voltage of 1 V was used to deposit PPy with a running time of 1500 s. After polymerization, the PPy@CMF was cleaned with distilled water three times.

The obtained PPy@CMF was carbonized at 300 °C for 1 h and 700 °C for 1 h under nitrogen atmosphere. The resulting electrode was denoted as NC@CMF.

### 2.5. MFC Set Up and Operation

All of the CMFs were cultured in an H-shaped dual-chamber reactor with a volume of 100 mL for every chamber. A carbon brush was used as the cathode, 50 mM potassium ferricyanide and potassium chloride mixed solution was used as the catholyte. The anolyte contained 1 g sodium acetate, 500 mL phosphate-buffered solution (11.55 g L^−1^ Na_2_HPO_4_·12H_2_O, 2.77 g L^−1^ NaH_2_PO_4_·H_2_O, 0.31 g L^−1^ NH_4_Cl, and 0.13 g L^−1^ KCl), 5 mL trace mineral solution, and 500 μL vitamin (the formula of trace mineral solution and vitamin is shown in Table S1). The inoculum of the anode was sludge sediment of a second settling tank gathered from the Taiping wastewater treatment plant in Harbin.

CC (2 cm × 3 cm, determined by the surface area of the CMF and NC@CMF), CMF, and NC@CMF (cube with 1 cm × 1 cm × 1 cm) were used as anodes. Sludge (20 mL) and anolyte (80 mL) were added to the anode chamber, which was aerated by N_2_ for 20 min, then 100 mL catholyte was added to the cathode chamber. The culture temperature was maintained at 37 °C and a 1000 Ω resistor was used as the circuit load. The medium was replaced when the voltage was decreased to lower than 50 mV.

### 2.6. Electrochemical Characterizations

Cyclic voltammetry (CV) and differential pulse voltammetry (DPV) were conducted on the electrochemical workstation (CHI 760E, Chenhua, Shanghai, China). Electrochemical impedance spectroscopy (EIS) was conducted on the impedance/gain-phase analyzer (1260, Solartron Metrology Inc., Gastonia, NC, USA). All of the tests were collected in a three-electrode cell, which included an anode as working electrode, a saturated calomel electrode (SCE), and a platinum plate counter electrode. CV was recorded in the anolyte between −0.8 and 0.2 V with a scan rate of 5 mV s^−1^. DPV was measured from −0.6 to 0.4 V in the anolyte with amplitude 60 mV, potential increment 6 mV, and pulse width 200 ms. EIS was tested in the anolyte for the frequency range between 100 kHz and 0.1 Hz with a direct current potential of 0 V versus open-circuit potential (OCP) and alternating voltage amplitude of 30 mV.

### 2.7. Microbial Community Analysis

After 60 days, the MFC anodes with biofilms were taken out and washed three times in PBS solution. The DNA of the biofilms on the anodes was extracted using the Power Soil DNA Isolation Kit and stored at −20 °C for testing. The microbial community was analyzed via high-throughput 16S rRNA gene pyrosequencing.

## 3. Results and Discussions

### 3.1. Synthesis and Characterization of Anodes

**Figure 1:**
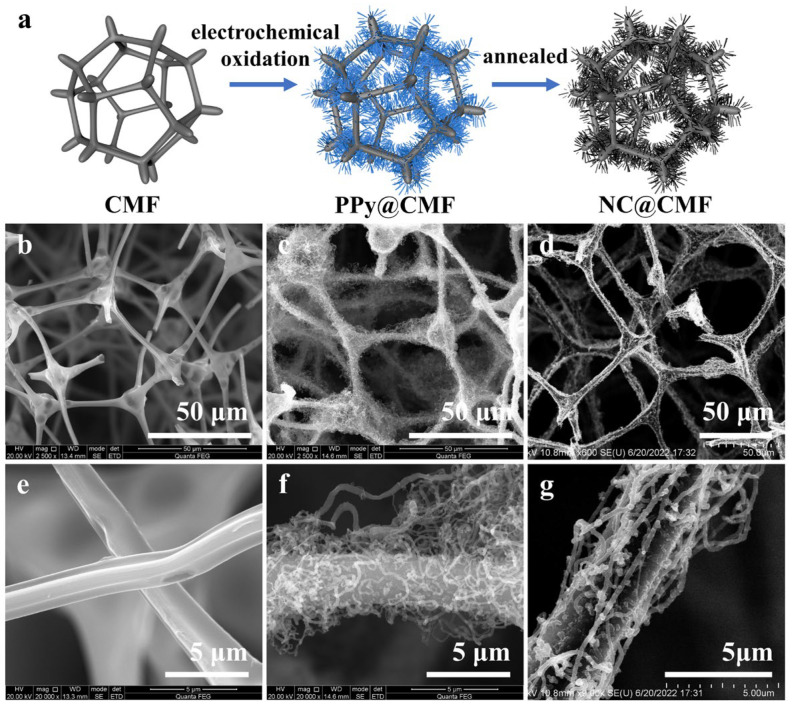
(**a**) Schematic procedure of anode preparation. The low- and high-resolution SEM images of (**b**,**e**) CMF, (**c**,**f**) PPy@CMF and (**d**,**g**) NC@CMF.

As shown in Figure 1a, 3D porous carbonized melamine foam was used as the working electrode to electrodeposit PPy, then annealed at 300 °C for 1 h and 700 °C for 1 h under a N_2_ atmosphere to convert PPy into N-doped carbon nanowires. SEM was used to observe the changes in the surface micro-/nanostructure of the material during the preparation process. As shown in Figure 1b,e, CMF consists of a 3D interlocking macroporous structure with a pore size of 50–100 µm. After the electrochemical deposition process, PPy nanowires grew uniformly on the carbon skeleton (Figure 1c,f), with an average diameter of ca. 150.6 nm. Figure 1d,g show that during the carbonization process, the structure of N-doped carbon nanowires was well preserved, and their diameter increased to ca. 212.8 nm. On the one hand, N-doped carbon nanowires will serve as artificial conductive wires, connecting bacteria with other remote bacteria to ensure the long-distance transfer of electrons generated by the outer layer of the biofilm [29,30]. On the other hand, pyrrolic N can absorb electrons from outer membrane c-Cyts (OMCs) and flavin by reducing the binding energy and shortening the electron transfer distance [31]. To demonstrate the presence of N, we performed element mapping of CMF and NC@CMF. As shown in Figure S1, the N element was uniformly distributed on the surface of the skeleton structure of the two anodes.

**Figure 2:**
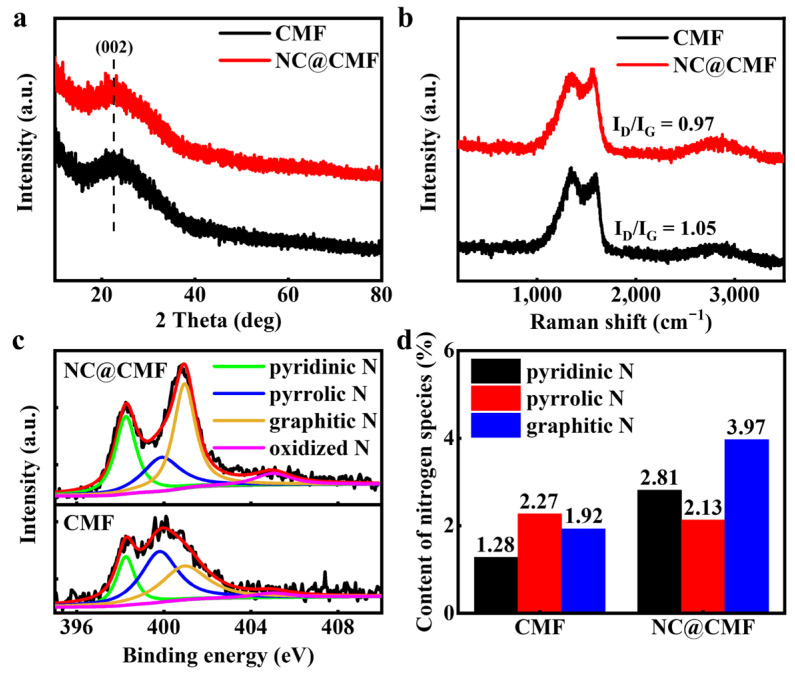
Characterization of CMF and NC@CMF: (**a**) XRD patterns, (**b**) Raman spectra, (**c**) N1s XPS spectra, and (**d**) the histogram of different N species contents.

As shown in Figure 2a, CMF and NC@CMF display a wide diffraction peak at 2θ = 23°, which was identified as the (002) crystal plane of carbon (PDF No. 50-0926). To verify the degree of carbonization, CMF and NC@CMF were subjected to Raman spectroscopy. The Raman spectrum in Figure 2b shows that the I_D_/I_G_ value of CMF is 1.05. After modifying the N-doped carbon nanowire, the I_D_/I_G_ value decreases to 0.97, indicating an increase in graphitization degree, which is beneficial for improving the intrinsic conductivity of the anode and promoting electron transport within the 1D nanowire [32]. To analyze N species, we tested XPS full spectra (Figure S2a) and fine spectra of various elements for CMF and NC@CMF. Both materials contain C, N, and O elements. Figure 2c shows the XPS deconvolution spectra of N 1s, with peaks at binding energies of 398.25, 399.9, 400.95, and 405 eV, which are attributed to pyridinic N, pyrrolic N, graphitic N, and oxidized N [33], respectively. Figure 2d shows the content of N species, indicating the total N content is 9.68% in NC@CMF, higher than that of CMF (5.84%). The sum of pyrrolic N and graphitized N is ca. 6.1% in the former anode, which is higher than that in the latter one (4.19%). The previous literature confirmed that these two N species can optimize the adsorption energy of hemin on their surfaces, shorten the electron transfer distance, and increase the EET rate [33]. On the other hand, doping with N atoms can improve the hydrophilicity of carbon materials [34,35]. As shown in Figure S3, the contact angle of CMF is 131.75°. However, the contact angle of NC@CMF decreases to 0°, which may also be related to the capillary effect of the surface nanowires. The hydrophilic electrode surface can promote the infiltration of anode solution into the interior of the electrode in the early stage, and N doping can induce the localization of positive charges on the surface of carbon materials, which synergistically promotes the growth of negatively charged microorganisms [36]. Further comparing the C 1s deconvolution spectra in Figure S2b, it was found that the proportion of graphitized carbon in NC@CMF is 65.1%, higher than that of CMF (51.9%), which is consistent with Raman spectroscopy results, indicating that the degree of graphitization of modified nanowires increases, which is beneficial for reducing material resistance.

**Figure 3:**
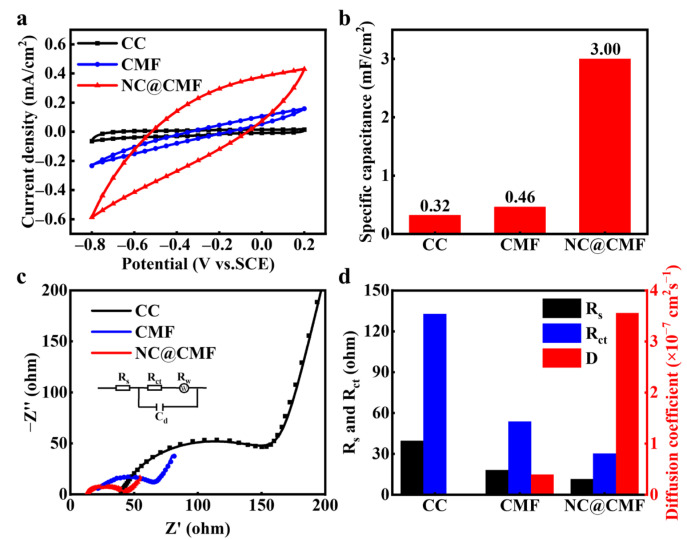
Electrochemical properties of different anodes: (**a**) CV and (**b**) specific capacitance. (**c**) EIS and (**d**) *R_s_*, *R_ct_* and the ion diffusion coefficient (*D*).

The electrochemical activity and specific capacitance of the anode material were characterized using cyclic voltammetry (CV) in the anolyte. As shown in Figure 3a, there is no obvious redox peak on the curve, indicating that the material has no catalytic activity against the acetate substrate. By calculating the area of the CV curve, it can be seen that NC@CMF exhibits higher current density and larger specific capacitance than CC and CMF. As shown in Figure 3b, the specific capacitance of NC@CMF is 3 mF/cm^2^, which is 6.52 and 9.38 times that of CMF and blank CC, respectively. This indicates that the former can store more electrons generated by microbial metabolism, which is beneficial for improving production capacity and COD removal ability [37,38]. Generally speaking, the EET process is partially limited by the internal resistance of the anode, so we compared the EIS data of three electrodes. In the Nyquist curves, the anode appears as a semicircle in the high-frequency range, and the diameter of the semicircle is related to the charge transfer resistance (*R_ct_*). After fitting, as shown in Figure 3d, The *R_ct_* value of the NC@CMF electrode is 30.17 Ω, which is smaller than that of CMF (53.67 Ω) and CC (132.6 Ω). A smaller *R_ct_* means less energy loss during the EET process, which is beneficial for improving the output power of the battery [39,40]. The *D* of three materials can be obtained by fitting the straight lines within the low-frequency range of the Nyquist plots. The *D* value of NC@CMF is 3.56 × 10^−7^ cm^2^ s^−1^, about one and three orders of magnitude higher than that of CMF (3.96 × 10^−8^ cm^2^ s^−1^) and CC (2.75 × 10^−10^ cm^2^ s^−1^). In general, the higher the *D* value, the better the mass transfer ability of the anode, which can promote the diffusion of acetate ions and improve the metabolic activity of bacteria [41].

**Figure 4:**
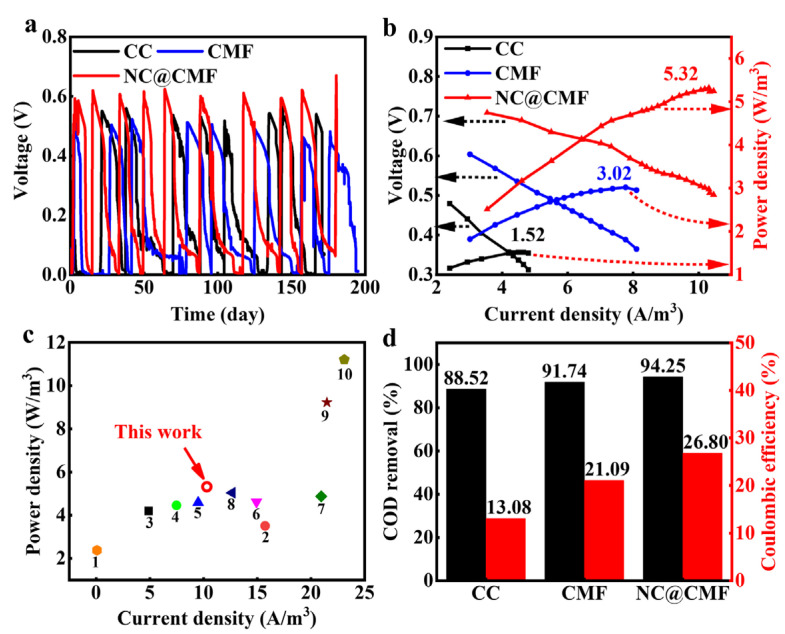
The performance of MFCs with CC, CMF, NC@CMF as anodes: (**a**) The output voltage curves. (**b**) Polarization curves and power density. (**c**) Comparisons with the previously reported 3D anodes (1: GA [42]; 2: A-CMC-Gr-PD [43]; 3: CS-NCNT-PANI [44]; 4: HA/GA [45]; 5: G-800 [46]; 6: NP/SCC [47]; 7: PPy-CMC/N-CNT/S [48]; 8: N-MWCNT/GA [14]; 9: NPVP-RFC [21]; 10: HPCF [26]). (**d**) COD removal rate and coulombic efficiency. Different colors were used to represent different anodes accordingly.

Through the above characterization analysis, it is found that NC@CMF has various properties required for MFC anodes, such as a high content of pyrrolic and pyridinic N heteroatoms, evenly wrapped nanowires on a 3D skeleton, good wettability, high specific capacitance, low *R_ct_*, and large *D*, which may result in excellent MFC performance. A dual-chamber MFC structure was adopted with sodium acetate as the fuel, and the long-term cycling curve of the CC, CMF, and NC@CMF anodes were tested for nearly 180 days. As shown in Figure 4a, the MFC voltage of NC@CMF can stably output at 623 mV, higher than bare CC (564 mV) and CMF (530 mV). Figure 4b shows that the maximum output power density of NC@CMF is 5.32 W/m^3^, which is about 1.76 times higher than CMF (3.02 W/m^3^) and 3.5 times higher than CC (1.52 W/m^3^). We compared the power density of similar recently published anode materials in Figure 4c, and found that the NC@CMF anode was inferior to NPVP-RFC (9.23 W/m^3^) [21] and HPCF (11.21 W/m^3^) [26], but also superior to many similar materials, such as GA (2.38 W/m^3^) [42], A-CMC-Gr-PD (3.51 W/m^3^) [43], and CS-NCNT-PANI (4.2 W/m^3^) [44]. The NC@CMF anode possessed three advantages compared to other anodes. Its macroporous structure could ensure the mass transport and metabolic activity of bacteria, improving the EET process accordingly. NC on CMF favored the attachment of microorganisms, and provided abundant physical contact sites for bacteria, thus facilitating long-distance electron transfer in biofilm. In addition, pyrrolic N could optimize the binding energy of hemin and effectively shorten the electron transfer distance, then promoted the electron transfer from RF and OMCs to anodes. To sum up, NC@CMF anode exhibited a higher power density compared to bare CC or CMF without nanomaterials modification. The removal rate of sodium acetate was evaluated by using the Chemical Oxygen Demand (COD) removal method. As shown in Figure 4d, the COD removal rate of NC@CMF is 94.25%, higher than that of CMF (91.47%) and CC (88.52%). The coulombic efficiency of the NC@CMF anode reaches 26.8%, which is higher than that of CMF (21.09%) and CC (13.08%), indicating that NC@CMF can efficiently oxidize sodium acetate and convert chemical energy into electrical energy.

### 3.2. MFC Performance

**Figure 5:**
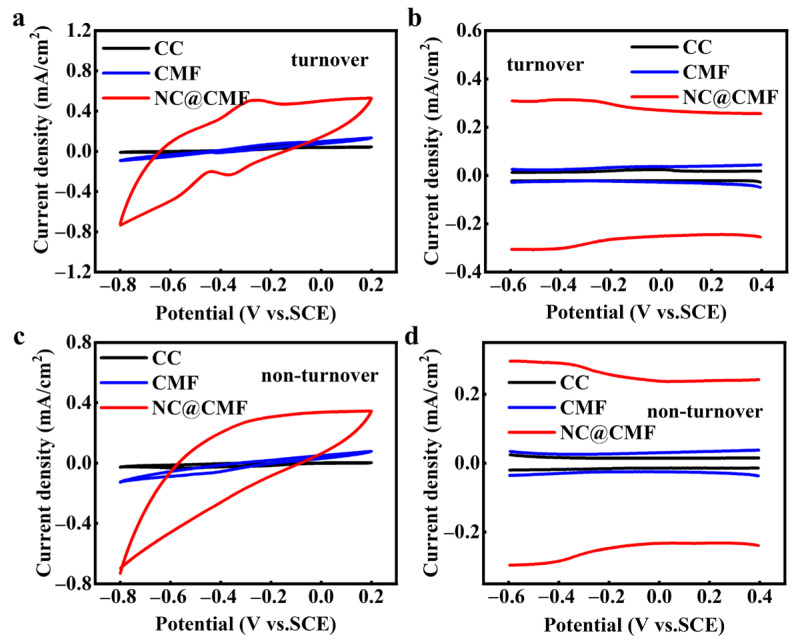
CV curves and DPV curves of different anodes under (**a**,**b**) turnover and (**c**,**d**) non-turnover conditions.

The performance of MFCs is directly related to the electrochemical performance of the biofilm on the anode surface. In order to study the electrochemical activity of biofilms, the CV and DPV curves of biofilms on three anodes were tested. Under turnover conditions, the anolyte contains sufficient sodium acetate to characterize the oxidation ability of the biofilm. Figure 5a,b show that the maximum specific current of NC@CMF (0.508 mA/cm^2^) was 5.91-fold and 8.47-fold that of CMF (0.086 mA/cm^2^) and CC (0.060 mA/cm^2^), demonstrating much higher catalytic activity for sodium acetate. Under non-turnover conditions, namely where sodium acetate is depleted, the oxidation–reduction process of OMCs on the bacterial outer membrane and flavin shuttles can be characterized. As shown in Figure 5c,d, the NC@CMF anode exhibited a shoulder peak and a wide peak around −0.15 V and −0.45 V, attributed to the free OMCs and the binding of flavin with OMCs [49,50]. These results indicated that the NC@CMF anode had a better EET ability than the control groups.

**Figure 6:**
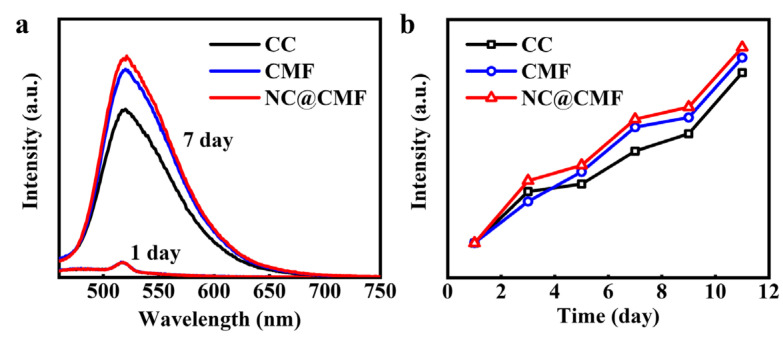
Flavin excretion in various anodes: (**a**) Fluorescence emission spectra of the three anolytes without addition of flavin at 1st and 7th day. (**b**) Corresponding emission spectra intensity of the three anolytes in 11 days.

In order to prove that NC@CMF could promote the excretion of flavin by bacteria, a fluorescence emission spectrum was used to measure the content of flavin in freshly assembled MFCs in Figure S4. Figure 6a shows that the flavin contents of the three MFCs were very small on the first day, indicating that only a small amount flavin molecules were generated after 24 h inoculation. In sharp contrast, after a week, the flavin content in the anolyte containing the NC@CMF anode was higher than that of the other two anodes of CMF and CC. In Figure 6b, the growth curves of flavin content in 11 days are recorded. During the whole stage, NC@CMF could drive more flavin excretion than the control groups. In general, flavin can transfer electrons to the anode through remote diffusion, thus the increase in flavin content can promote the EET process in bacteria [51].

**Figure 7:**
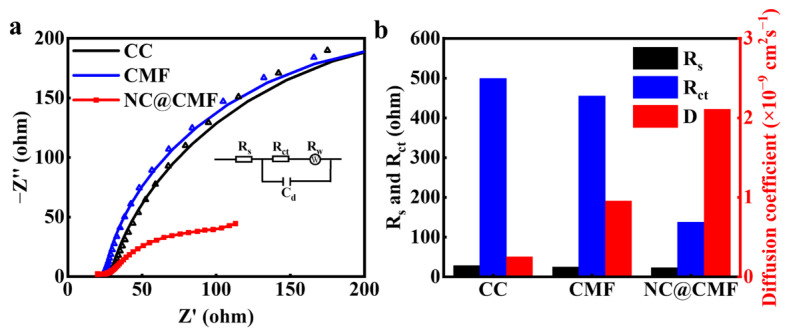
(**a**) Nyquist plots in anolyte and (**b**) *R_s_*, *R_ct_*, and *D* values of various anodes with biofilms.

After inoculation for a certain time, mature biofilms were grown on the anodes. The electrochemical behavior was changed accordingly. Thus, the resistance and mass transfer ability of different anodes were further estimated using EIS. As shown in Figure 7a,b, the *R_s_* of NC@CMF was 22.90 Ω, smaller than that of CMF (24.17 Ω) and CC (28.01 Ω). And the *R_ct_* of NC@CMF was 137.2 Ω, which was also the lowest among the other anodes (455.1 Ω for CMF and 499.1 Ω for CC). In addition, the *D* value of NC@CMF (2.11 × 10^−9^ cm^2^ s^−1^) was higher than that of CMF (9.56 × 10^−10^ cm^2^ s^−1^) and CC (2.54 × 10^−10^ cm^2^ s^−1^). Thus, the NC@CMF anode reduced *R_s_*, *R_ct_*, and *D*, which improved the conductivity and diffusion of the biofilm, leading to a faster EET rate.

**Figure 8:**
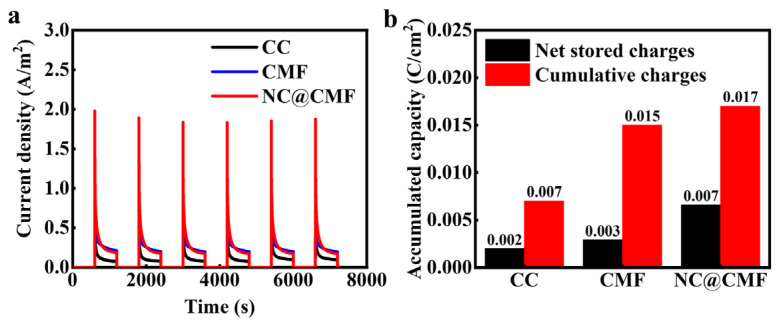
(**a**) Charging and discharging curves for CC, CMF, and NC@CMF. (**b**) Corresponding accumulated charge capacity.

According to the CV curves, it is seen that NC@CMF showed relatively larger specific capacitance in comparison with the other control groups. Thus, a charging and discharging test of biofilms on different anodes was carried out to evaluate their charge accumulation capacity. The MFC was disconnected and charged for 600 s with an open circuit. Then, the discharging test was conducted using the amperometric method with voltage of −0.4 V. The discharging time was set as 600 s. The same periods were applied for the charging and discharging processes. And the charging and discharging processes were performed for six cycles. Figure 8a,b show that the cumulative charges and net stored charges of NC@CMF were 0.017 C/cm^2^ and 0.007 C/cm^2^, which are higher than those of CMF (0.015 C/cm^2^ and 0.003 C/cm^2^) and CC (0.007 C/cm^2^ and 0.002 C/cm^2^). This result means that the biofilms of NC@CMF can store more electrons produced by bacteria.

**Figure 9:**
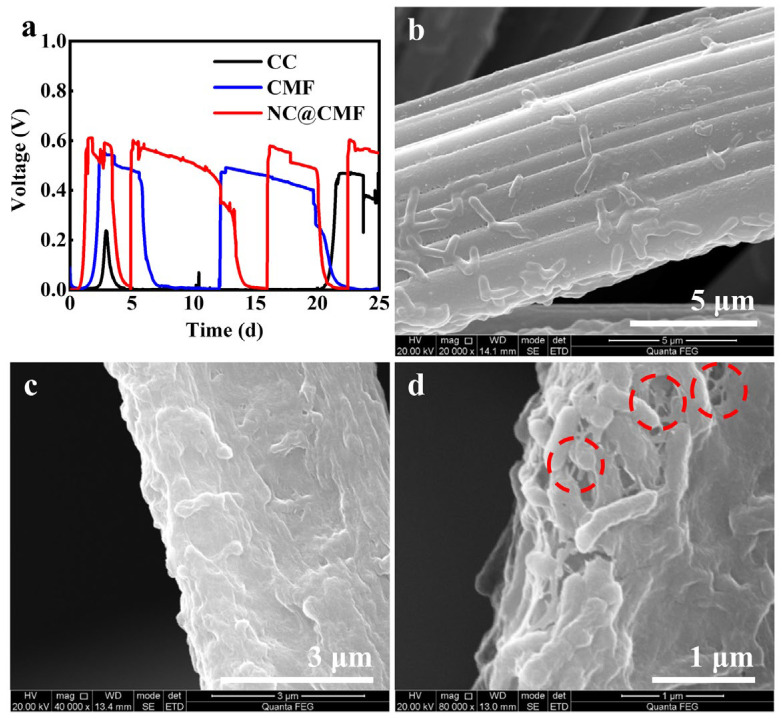
The performance of MFCs with CC, CMF, NC@CMF as anodes during start-up period: (**a**) The output voltages. The SEM images of biofilms on (**b**) CC, (**c**) CMF, (**d**) NC@CMF anodes. Red circles in (**d**) indicated the presence of nanoconduits.

To evaluate the bacteria capture efficiency of the anodes during the start-up period, SEM was performed after the start-up. Figure 9a shows the V-T curves at the start-up period. It is seen that the start-up time of NC@CMF was ca. 1.2 days, while that of CMF and CC was 2.3 days and 21 days, respectively. The SEM image in Figure 9b shows that a small amount of bacteria covered the CC. However, thick biofilms in Figure 9c,d can be observed on the CMF and NC@CMF anodes, further showing that macroporous structure favored bacterial adhesion. In addition, as indicated by red circles in Figure 9d, a large number of nanoconduits were found on the NC@CMF anode, which served as connector for the bacteria and the anode surface, as well as for different bacteria, thus accelerating the interspecies electron transfer and abiotic–biotic EET.

### 3.3. Biofilm Activity and Microbial Community Evaluation

**Figure 10:**
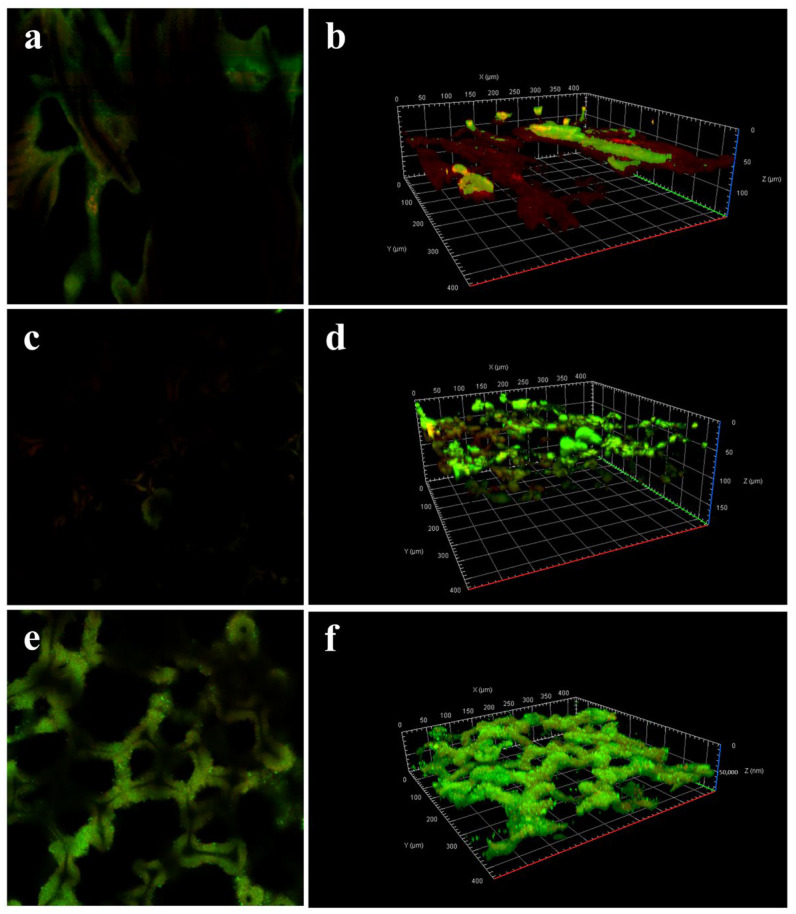
CLSM photographs of (**a**,**b**) CC, (**c**,**d**) CMF, and (**e**,**f**) NC@CMF biofilms after 60 days.

SEM images were acquired after 60 days inoculation for all anodes. As seen in Figures S5 and S6, mature biofilms were found on both the outer surface and inside of the anodes. According to the metabolism behavior of microorganisms, nutrients and waste inside a biofilm both need 3D transfer paths. However, commercial CC lacks a macroporous 3D structure. As expected, the CLSM image in Figure 10a,b displays that a large portion of red color was observed, which resulted from dead microorganism due to the issue of limited diffusion [52]. In sharp contrast, both CMF and NC@CMF exhibited stronger green fluorescence, indicating a higher proportion of live bacteria. In addition, a relatively thick biofilm was found on NC@CMF compared with CMF, ascribed to its better biocompatibility. In order to further prove the microorganism activity inside the anode, CLSM images of the biofilm inside NC@CMF and CMF are also measured in Figure S7. Some yellow fluorescence over the inside surface of CMF was found in CMF, which was related to a partially apoptotic microorganism. However, green fluorescence was maintained over the inside surface of both anodes, further proving the superiority of nanowire-modified 3D macroporous anodes.

**Figure 11:**
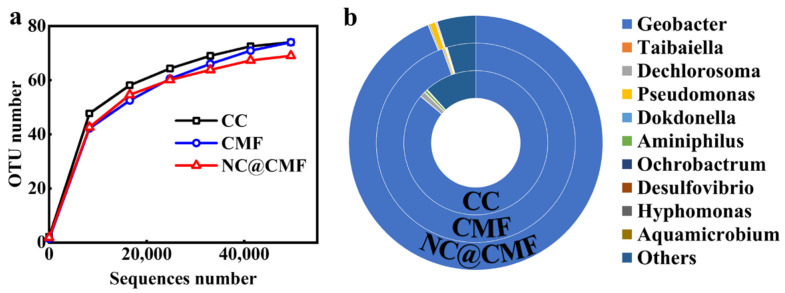
(**a**) Rarefaction curve of microbial community colonized on different anodes. (**b**) Distribution of microbial community at different anodes.

We further analyzed the microbial community structure of three anodes through 16S rRNA gene sequencing. Figure 11a shows the operational taxonomic unit (OUT) numbers. The curve approaching flatness means that the sequencing data were sufficient to represent the entire microbial community [53]. The analysis results of genus level are shown in Figure 11b. The content of *Geobacter* as an electroactive bacterium in the biofilm on NC@CMF was 94%, equal to that of CMF (94%) and higher than that of CC (84%). The enrichment of *Geobacter* was probably related to the 3D macroporous structure and its N-doped carbon surface. The content of electroactive bacteria directly affected the output voltage and power performance of the MFCs [54].

### 3.4. Mechanism Analysis

**Figure 12:**
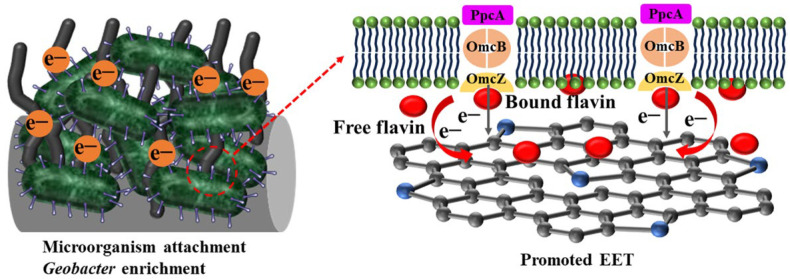
Schematic illustration of mechanism.

As shown in Figure 12, the excellent performance of the NC@CMF anode in MFCs is mainly attributed to the following aspects. Firstly, narrow pores exist in commercial CC, carbon felt (CF), or carbon paper (CP). In NC@CMF, nanowire-modified carbonized melamine maintains a 3D open macroporous structure, which is conducive to mass transfer and diffusion during long-term operation. Acetate can easily diffuse to the biofilm inside the anode, and metabolic waste is also easily removed, which can maintain the long-term stability of mature biofilms. This can be confirmed by our long-term stable experimental data of 180 days, and CLSM images have also confirmed that the biofilm activity is very high. Secondly, the structure of N-doped carbon nanowire-modified macroporous anodes can compensate for their poor biocompatibility. From the battery data of CMF, it can be seen that its output voltage is not high, but after N-doped carbon nanowire modification, the voltage increases to 623 mV. N doping can improve the wettability of the anode and also generate localized positively charged regions in the carbon skeleton structure, attracting negatively charged bacteria to adhere. The high sp^2^-hybridized C content in NC@CMF is also beneficial for reducing charge transfer resistance during the EET process. XPS data also confirm that our anode material contains high levels of graphitized N and pyrrolic N, and sp^2^-hybridized C. Previous DFT calculations have predicted that such N species can promote the adsorption of hemin or flavin, shorten the electron transfer distance, facilitate electron transfer at the interface, and improve the anode’s electrochemical performance [33,35,55]. Finally, the monitoring experiment of flavin confirmed that NC@CMF can promote its excretion, and a large amount of flavin can not only serve as an electron mediator to promote indirect electron transfer, but can also form complexes with hemin in bacterial OMCs, promoting DET and synergistically increasing the EET rate. Through RNA sequencing analysis of microbial communities, it was found that *Geobacter* can be enriched in NC@CMF. A large number of mature, electricity-generating, and highly active biofilms adhere to the anode surface, efficiently metabolizing acetic acid in the anolyte while also improving the power generation capacity and sewage treatment capacity of the NC@CMF anode.

## 4. Conclusions

In summary, a carbonized melamine self-supporting MFC anode modified with N-doped carbon nanowires was prepared through electrochemical oxidation polymerization and high-temperature carbonization strategies. This nanowire-modified 3D macroporous foam structure has good biocompatibility, which is conducive to bacterial adhesion and enrichment and the growth of high-activity biofilm. Highly conductive carbon nanowires, as artificial nanowires, build abundant physical contact sites between biofilms and anodes. In addition, the active sites generated by graphitized N- and pyrrolic N-doped atoms can accelerate the EET process by shortening the electron transfer pathway and reducing the binding energy. Nearly half a year of operational experiments have confirmed that the NC@CMF anode achieved better power output and COD removal rate, with a maximum platform voltage of 623 mV and a maximum power density of 5.32 W/m^3^, achieving a COD removal rate of 94.25%. The idea of 1D nanomaterial-modified macroporous electrodes developed in this work provides a feasible research approach for improving the power generation capacity of MFCs and wastewater treatment.

## Data Availability

Data available on request due to restrictions, e.g., privacy or ethical.

## Supplementary Materials

The following supporting information can be downloaded at: https://www.mdpi.com/article/10.3390/ma17010069/s1, Figure S1: The corresponding EDS elemental mapping of C, N, and O on (a–c) CMF and (d–f) NC@CMF; Figure S2: (a) XPS full spectra and (b) C1s XPS spectra of CMF and NC@CMF; Figure S3: The contact angle test of CMF and NC@CMF; Figure S4: Emission spectra of (a) CC, (b) CMF, and (c) NC@CMF after inoculation for 11 days; Figure S5: The SEM images of biofilms on the outside of (a,b) CC, (c,d) CMF, and (e,f) NC@CMF after 60 days; Figure S6: The SEM images of biofilms on the inside of (a,b) CMF and (c,d) NC@CMF after 60 days; Figure S7: CLSM photographs of biofilms on the inside of (a,b) CMF and (c,d) NC@CMF after 60 days; Table S1: Composition of minerals and vitamins in anolyte; Table S2: Reported 3D porous anodes compared with NC@CMF. Ref. [56] can be found in Supplementary Materials.
