# Supplementary material for: N-Doped Carbon Nanowire-Modified Macroporous Carbon Foam Microbial Fuel Cell Anode: Enrichment of Exoelectrogens and Enhancement of Extracellular Electron Transfer
Source: Materials (Basel). 2023 Dec 22;17(1):69. doi: 10.3390/ma17010069 (PMC10779606; doi:10.3390/ma17010069)
Supplement: Supplementary file 1 [file materials-17-00069-s001.zip › materials-2740724-supplementary.pdf]

Supporting Information

N-Doped Carbon Nanowire-Modified Macroporous Carbon Foam Microbial Fuel Cell Anode: Enrichment of Exoelectrogens and Enhancement of Extracellular Electron Transfer

Ke Liu [1], Zhuo Ma [2], Xinyi Li [3], Yunfeng Qiu [3,*], Danqing Liu [1,*] and Shaoqin Liu [3]

[1] School of Material Science and Chemical Engineering, Harbin University of Science and Technology, Harbin 150040, China
[2] Harbin Institute of Technology, School of Life Science and Technology, Harbin 150001, China
[3] Key Laboratory of Microsystems and Microstructures Manufacturing, Harbin Institute of Technology, School of Medicine and Health, Harbin 150080, China
* Correspondence: qiuyf@hit.edu.cn (Y.Q.); danqingliu76@163.com (D.L.)

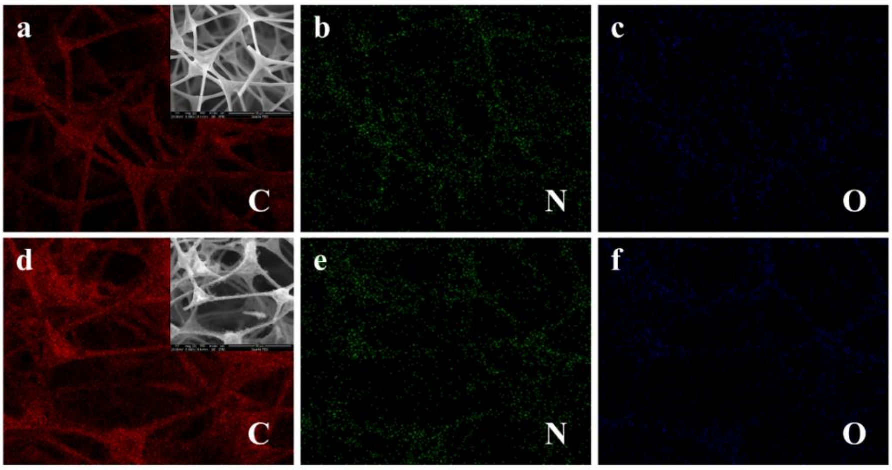

Figure S1. The corresponding EDS elemental mapping of C, N, and O on (a, b, and c) CMF and (d, e, and f) NC@CMF.

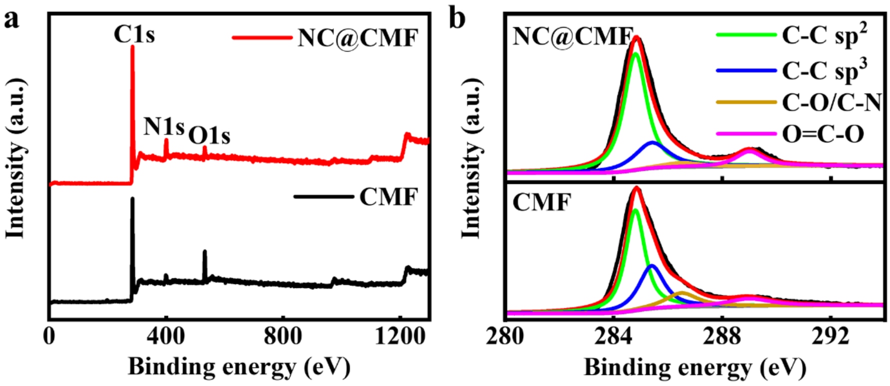

Figure S2. (a) XPS full spectra and (b) C1s XPS spectra of CMF and NC@CMF.

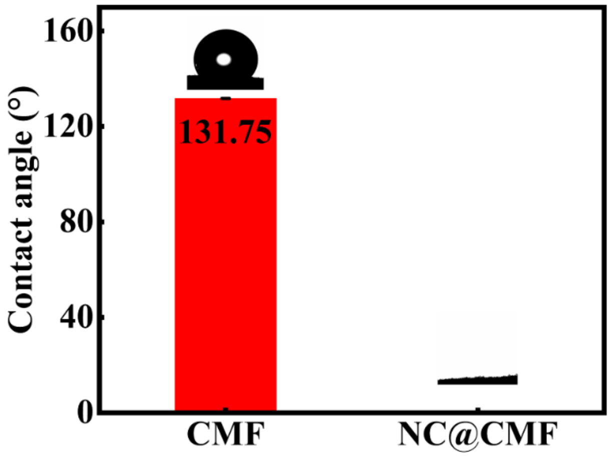

Figure S3. The contact angle test of CMF and NC@CMF.

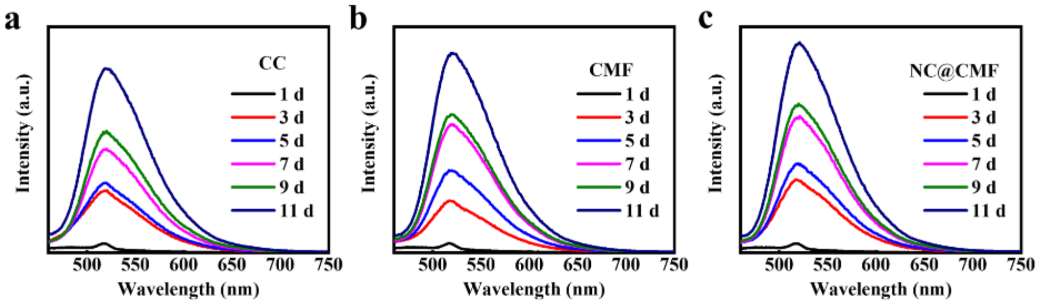

Figure S4. Emission spectra of (a) CC, (b) CMF, and (c) NC@CMF after inoculation for 11 days.

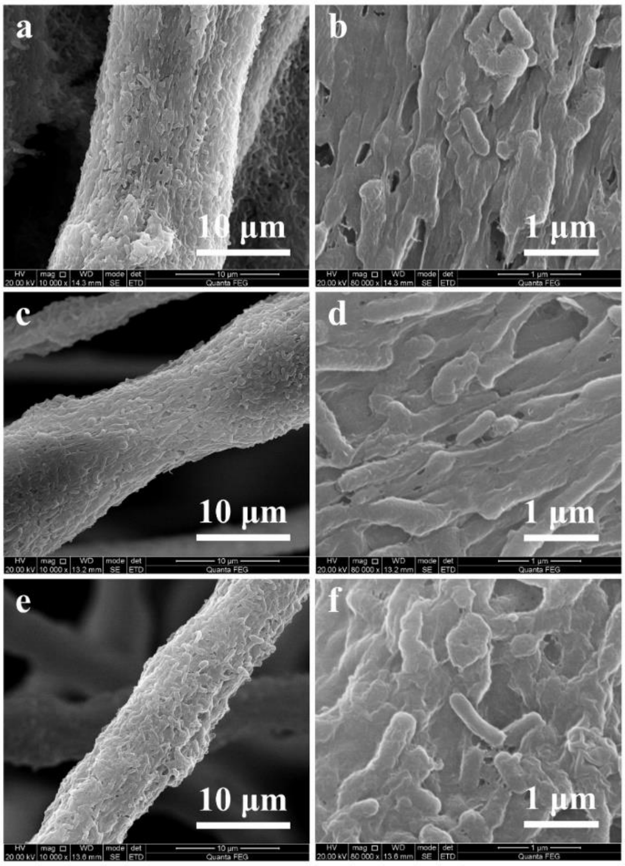

Figure S5. The SEM images of biofilms on the outside of (a, b) CC, (c, d) CMF, and (e, f) NC@CMF after 60 days.

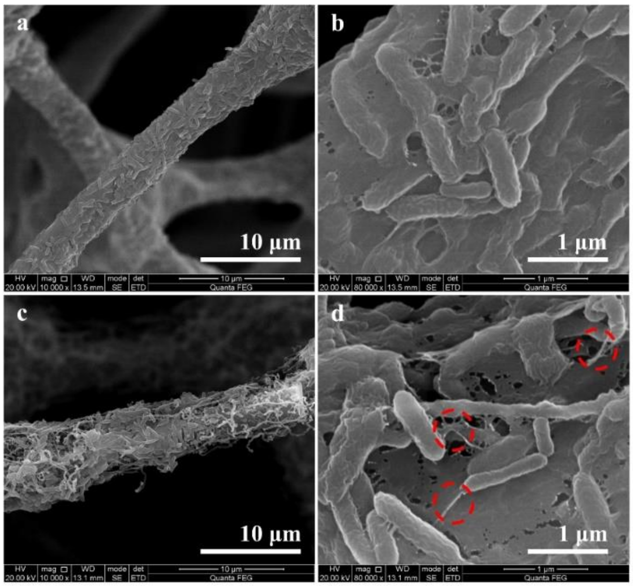

Figure S6. The SEM images of biofilms on the inside of (a, b) CMF and (c, d) NC@CMF after 60 days. Red circles in (d) indicated the presence of nanoconduits.

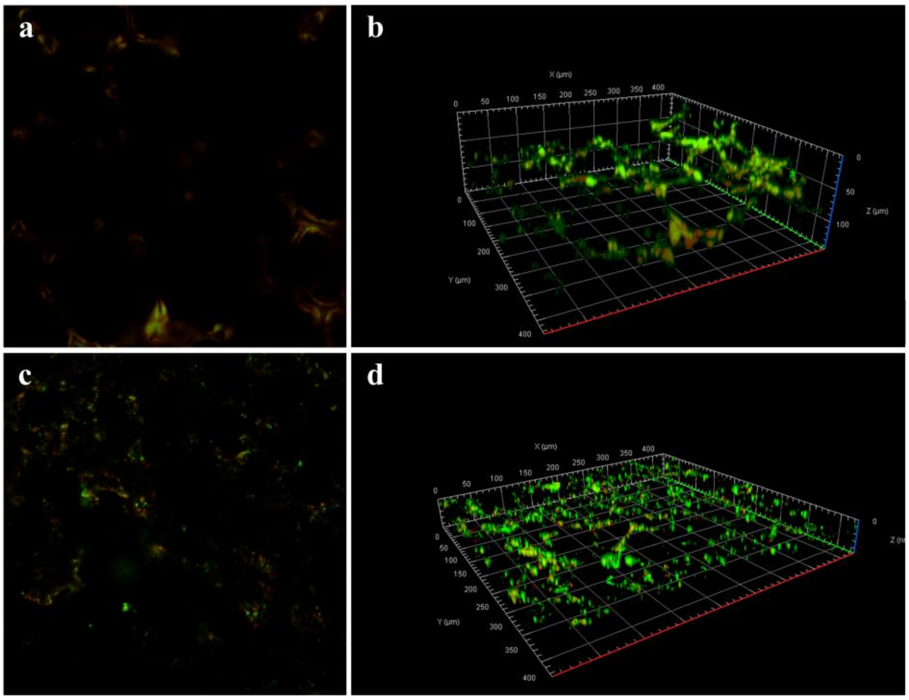

Figure S7. CLSM photographs of biofilms on the inside of (a, b) CMF and (c, d) NC@CMF after 60 days.

Table S1. Composition of minerals and vitamins in anolyte.

Vitamins (μg L^{-1})		Minerals (mg L^{-1})	
biotin	2	$MgSO_4{\cdot}7H_2O$	30
folic acid	2	NTA	15
p-aminobenzoic acid	5	NaCl	10
thioctic acid	5	$FeSO_4{\cdot}7H_2O$	1
riboflavin	5	$CuSO_4{\cdot}5H_2O$	0.1
aneurine HCl	5	$CoCl_2{\cdot}6H_2O$	1
nicotinic acid	5	$CaCl_2$	1
calcium pantothenate	5	$ZnSO_4{\cdot}7H_2O$	1
B–12	0.1	$Na_2MoO_4{\cdot}2H_2O$	0.1
pyridoxine HCl	10	H_3BO_3	0.1
		$AlK(SO_4)_2{\cdot}12H_2O$	1
		$MnSO_4{\cdot}H_2O$	5

Table S2. Reported 3D porous anodes compared with NC@CMF.

Anode material	Maximum volume power density* (W/m^3)	Inoculum	Substrate	References
GA	2.38	Mixed culture	Sodium acetate	[1]
A-CMC–Gr–PD	3.51	Mixed culture	Sodium acetate	[2]
CS-NCNT-PANI	4.2	Mixed culture	Sodium acetate	[3]
HA/GA	4.46	Mixed culture	Sodium acetate	[4]
G-800	4.59	Mixed culture	Sodium acetate	[5]
NP/SCC	4.62	Mixed culture	Sodium acetate	[6]
PPy-CMC/N-CNT/S	4.88	Mixed culture	Sodium acetate	[7]
N-MWCNT/GA	5.04	Mixed culture	Sodium acetate	[8]
NPVP-RFC	9.23	Mixed culture	Sodium acetate	[9]
HPCF	11.21	Mixed culture	Sodium acetate	[10]
NC@CMF	5.32	Mixed culture	Sodium acetate	**this work**

* Power density was normalized by anode chamber volume.

References

1. Yu, F.; Wang, C.; Ma, J. Capacitance-Enhanced 3D Graphene Anode for Microbial Fuel Cell with Long-Time Electricity Generation Stability. *Electrochim. Acta* **2018**, *259*, 1059–1067.
2. Call, T.P.; Carey, T.; Bombelli, P.; Lea-Smith, D.J.; Hooper, P.; Howe, C.J.; Torrisi, F. Platinum-Free, Graphene Based Anodes and Air Cathodes for Single Chamber Microbial Fuel Cells. *J. Mater. Chem. A* **2017**, *5*, 23872–23886.
3. Xu, H.; Wang, L.; Wen, Q.; Chen, Y.; Qi, L.; Huang, J.; Tang, Z. A 3D Porous NCNT Sponge Anode Modified with Chitosan and Polyaniline for High-Performance Microbial Fuel Cell. *Bioelectrochemistry* **2019**, *129*, 144–153.
4. Zhao, T.; Qiu, Z.; Zhang, Y.; Hu, F.; Zheng, J.; Lin, C. Using a Three-Dimensional Hydroxyapatite/Graphene Aerogel as a High-Performance Anode in Microbial Fuel Cells. *J. Environ. Chem. Eng.* **2021**, *9*, 105441.
5. Li, J.; Qiu, Y.; Li, D.; Wu, J.; Tian, Y.; Liu, G.; Feng, Y. Revealed Mechanism of Micron-Pore Size of 3d Bio-Anode on the Behavior of Biofilm and System Performance in Microbial Electrochemical System. *Chem. Eng. J.* **2023**, *464*, 142736.
6. Yuan, H.; Dong, G.; Li, D.; Deng, L.; Cheng, P.; Chen, Y. Steamed Cake-Derived 3D Carbon Foam with Surface Anchored Carbon Nanoparticles as Freestanding Anodes for High-Performance Microbial Fuel Cells. *Sci. Total Environ.* **2018**, *636*, 1081–1088.
7. Wang, Y.; Pan, X.; Chen, Y.; Wen, Q.; Lin, C.; Zheng, J.; Li, W.; Xu, H.; Qi, L. A 3D Porous Nitrogen-Doped Carbon Nanotube Sponge Anode Modified with Polypyrrole and Carboxymethyl Cellulose for High-Performance Microbial Fuel Cells. *J. Appl. Electrochem.* **2020**, *50*, 1281–1290.
8. Jin, S.; Feng, Y.; Jia, J.; Zhao, F.; Wu, Z.; Long, P.; Li, F.; Yu, H.; Yang, C.; Liu, Q.; et al. Three-Dimensional N-Doped Carbon Nanotube/Graphene Composite Aerogel Anode to Develop High-Power Microbial Fuel Cell. *Energy Environ. Mater.* **2022**, *6*, e12373.
9. Sun, J.; Wang, R.; Li, H.; Zhang, L.; Liu, S. Boosting Bioelectricity Generation Using Three-Dimensional Nitrogen-Doped Macroporous Carbons as Freestanding Anode. *Mater. Today Energy* **2023**, *33*, 101273.
10. Li, H.; Zhang, L.; Wang, R.; Sun, J.; Qiu, Y.; Liu, S. 3D Hierarchical Porous Carbon Foams as High-Performance Free-Standing Anodes for Microbial Fuel Cells. *EcoMat* **2022**, *5*, e12273.
